# Neuroprotective Effects of Ethiopian Coffee Beans against Hyperglycemia‐induced Brain Injury in Rats

**DOI:** 10.1002/fsn3.72054

**Published:** 2026-07-08

**Authors:** Almahi I. Mohamed, Kolawole A. Olofinsan, Ochuko L. Erukainure, Huda Ismail, Md. Shahidul Islam

**Affiliations:** ^1^ Department of Biochemistry, School of Agriculture and Science University of KwaZulu‐Natal, Westville Campus Durban South Africa; ^2^ Department of Pharmacology University of the Free State Bloemfontein South Africa

**Keywords:** Arabica coffee beans, diabetes, hyperglycemia, neurodegeneration, neuroprotection, oxidative stress

## Abstract

At least 50% of people with diabetes suffer from one or more complications if their conditions are not adequately managed over time. Diabetic neuropathy is one of the prevalent complications of diabetes, which also includes diabetic nephropathy, retinopathy, cardiomyopathy, and diabetic foot diseases. The present study evaluated the protective effects of Ethiopian coffee beans ( *Coffea arabica* ) against glucose‐induced brain tissue injury using in vitro, ex vivo, and in silico experimental models. Oxidative injury was induced by incubating brain tissue collected from normal male Sprague–Dawley rats in glucose solution and treated with the different concentrations of Ethiopian coffee bean extracts (hot and cold aqueous) for 2 h at 37°C in a 95% O_2_ and 5% CO_2_ incubator. Induction of glucose‐mediated (0.0111 M glucose) oxidative injury led to significant depletion of reduced glutathione (GSH), superoxide dismutase (SOD), catalase (CAT), and total glycogen levels, while elevating malonaldehyde (MDA), nitric oxide (NO), glycogen phosphorylase, fructose‐1,6‐bisphosphatase, ATPase, and acetylcholinesterase (AChE) activity levels. Treatment with different concentrations of the aqueous extracts of coffee beans significantly restored the levels and activities of the biomarkers mentioned above. LC–MS analysis indicates the presence of chlorogenic acid (CGA), caffeic acid, quinic acid, caffeine, Cafestol, Kahweol, ferulic acid, and catechol in the coffee extracts. In silico analysis revealed a strong molecular interaction between CGA and the CAT, SOD, and AChE enzymes. The data from this study suggest that bioactive compounds from *Coffea arabica* have a potential neuroprotective effect against glucose‐mediated oxidative neurodegeneration in rat brain tissue.

## Graphical abstract

Ethiopian coffee bean extracts, particularly the cold aqueous preparation, demonstrated significant neuroprotective effects against hyperglycemia‐induced oxidative brain injury in an ex vivo rat brain tissue model. The extracts mitigated oxidative stress by restoring antioxidant defenses (GSH, SOD, CAT) and reducing lipid peroxidation (MDA) and nitrosative stress (NO). Furthermore, they improved glucose metabolism by increasing glucose uptake and glycogen content while reducing the activity of key enzymes linked to neuronal dysfunction, including acetylcholinesterase (AChE) and ATPase. In silico analysis identified chlorogenic acid as a key bioactive compound, showing strong binding affinities with neuroprotective and metabolic enzymes (CAT, SOD, and AChE).

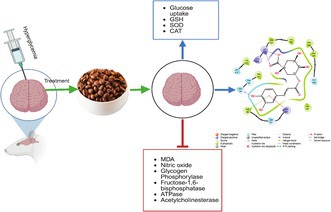

## 1 Introduction

Type 2 diabetes (T2D) is a disease condition with characteristics including hyperglycemia, insulin secretory inadequacy, or insulin receptor insensitivity (Kostov 2019). It is the most prevalent type of diabetes, accounting for more than 90% of all diabetes (Cole and Florez 2020). T2D is linked to both microvascular and macrovascular disorders that impact many different organs, causing neuropathy, cardiopathy, retinopathy, and nephropathy (Mota et al. 2020). The onset of these pathophysiologies is often associated with hyperglycemia, which develops from unregulated blood glucose levels (Alam et al. 2021).

Glucose is an indispensable energy source for the brain, as it is essential for its key processes, such as neuronal signaling, basic brain operations, and non‐signaling conductive functions (Banks 2019). The brain primarily relies on glucose as its energy source (Morea et al. 2017). During glucose transport to the brain, numerous interactions occur between substances, common carriers, enzymes, and cell signaling pathways within the complex structure of the blood–brain barrier (BBB) neurovascular unit (Gupta et al. 2019; Patching 2017). However, hyperglycemia in T2D mediates processes that impair BBB integrity and ultimately contribute to an increased risk of cerebral ischemia (Szeto et al. 2018). Since glucose is the primary energy source for the brain, impaired glucose transport across the BBB adversely affects brain cell function (Nimgampalle et al. 2021).

Type 2 diabetes (T2D) may be treated or managed with a broad range of commonly prescribed medications (Khunti et al. 2023). While representing one of these medications, metformin has been reported to have anti‐inflammatory and antioxidative effects on ischemic stroke that can affect the central nervous system (Sharma et al. 2021). Furthermore, metformin enhances glucose control in the brain through multiple mechanisms (Cao et al. 2022). Previous studies have demonstrated that metformin can cross the BBB and stimulate specific neurons and glial cells, producing neurological effects (Demaré et al. 2021; Leech et al. 2019). Nevertheless, despite the neuroprotective advantages of metformin, there is significant concern regarding its potential adverse effects on the brain and other organs (Li et al. 2022). However, medicinal plants and their bioactive phytochemicals have proven effective in treating chronic illnesses, including diabetes and its associated problems (Unuofin and Lebelo 2020). Coffee beans, as a medicinal plant part, have been reported to manage neurodegeneration complications related to diabetes (Al‐Brakati et al. 2020; Fatima et al. 2023).

Coffee has been reported to have antioxidants and anti‐diabetic properties (Socała et al. 2020). These health benefits have been associated with a wide range of phytochemicals, including phenolic acids, alkaloids, flavonoids, lipids, and proteins, present in the plant (Acidri et al. 2020). Moreover, the benefits of coffee compounds have been well established. They have been documented to act against oxidative stress, restore metabolic alterations, and ameliorate neurodegeneration (Colombo and Papetti 2020; Socała et al. 2020). There is evidence that coffee compounds, such as chlorogenic acid (CGA) and caffeine, may help mitigate neurodegeneration and related disorders associated with oxidative stress in the brain (Heitman and Ingram 2017; Oboh et al. 2013). Furthermore, numerous clinical and preclinical studies have demonstrated the beneficial impacts of coffee consumption on Alzheimer's disease and Parkinson's disease (Anggreani and Lee 2017; Wierzejska 2017).

The roasting process of coffee beans plays a crucial role in influencing their chemical composition and biological activities (Bolka and Emire 2020). This process ranges from mild to medium to dark roasting, each resulting in distinct changes in the biochemical composition and physiological functions of coffee bean components. Specifically, mild‐to‐medium roasting conditions, for instance at 150°C for 15 min, have been reported to preserve important antioxidant phytochemicals, including CGA, caffeic acid derivatives, and other phenolic compounds, while still promoting the formation of beneficial Maillard reaction products (Mohamed et al. 2024). Simultaneous preservation of these phytochemicals results in remarkable neuroprotective effects (Nabavi et al. 2017). In contrast, higher roasting temperatures can cause substantial degradation of CGA and other thermolabile compounds that contribute to coffee's biological activities, thereby reducing its antioxidant and anti‐diabetic capabilities (Nam and Kang 2015). Despite the reported anti‐diabetic and neuroprotective effects of coffee beans, there is currently a lack of information on how roasting temperature influences their ability to mitigate brain hyperglycemia and the neuroprotective mechanisms involved. Therefore, the present study was conducted to evaluate the effect of Ethiopian coffee ( *Coffea arabica* ) cold and hot extractions on metabolic enzyme alterations associated with hyperglycemia‐induced oxidative brain tissue damage, using both ex vivo and in silico approaches.

## 2 Material and Methods

### 2.1 Plant Sample

Ethiopian coffee beans (*Coffee arabica*) used in this study were harvested from the Harar coffee field in eastern Ethiopia in January 2025.

### 2.2 Roasting and Extraction of Coffee Beans

Coffee beans were roasted at 150°C for 15 min and subsequently ground into a fine powder following the traditional coffee preparation technique previously documented (Caprioli et al. 2015; Illy 2002). Hot and cold aqueous coffee extracts were then prepared. For the hot extraction, coffee powder was mixed with boiling distilled water (100°C), while for the cold extraction, the sample was mixed with distilled water at ambient temperature (23°C ± 2°C), which was sufficient to prevent the oxidative degradation of the extract's thermolabile phytochemicals. A coffee‐to‐water ratio of 1:10 (w/v) was used, whereby 40 g of ground coffee was extracted with 400 mL of distilled water (Mohamed et al. 2025). Both hot and cold preparations were macerated for 24 h. The resulting extracts were filtered and subsequently freeze‐dried before experimental analysis.

### 2.3 Preparation of Extraction Stocks

Stock solutions were prepared by dissolving the freeze‐dried Ethiopian coffee extracts in distilled water to obtain a final concentration of 1 mg/mL. Working solutions with 30, 60, 120, and 240 μg/mL concentrations were subsequently prepared from the stock solution and used for the ex vivo experimental analyses.

### 2.4 Animals Protocol

Six‐weeks old male Sprague–Dawley rats weighing 170 ± 20 g were obtained from the Biomedical Research Unit, University of KwaZulu‐Natal (UKZN), Durban, South Africa. The rats' treatment and administration adhered to the guidelines of the Animal Ethics Research Committee of the UKZN, with ethical approval number (AREC/00006156/2025). After the rats were sacrificed humanely, their brain tissues were immediately collected for ex vivo assays.

### 2.5 Assessment of Glucose Uptake in Rats' Brains

Using the protocol developed by Chukwuma and Islam (2015), the effect of the coffee bean aqueous extracts on glucose uptake in fresh brain tissues was assessed. Briefly, a 500 mg of brain tissue was transferred to an 8 mL solution of 0.0111 M glucose and 30–240 μg/mL of the extract in Krebs's buffer. Metformin (240 μg/mL) and a comparable glucose concentration in Krebs's buffer were utilized as the control for the study. The tissue solution was incubated at 37°C and 5% CO_2_ for 2 h. Before and after incubation, a 2 mL aliquot of the incubating solution was collected for glucose concentration measurement using an Automated Chemistry Analyzer (Labmax Plenno, Labtest Inc., Lagoa Santa, Brazil). The uptake of glucose by the isolated brain tissue was determined employing the following formula:

$$\text{Glucose uptake}\;\mathrm{per}\;\text{gram of}\;\mathrm{rat}\;\text{brain tissue}=\frac{\mathrm{GC}1-\mathrm{GC}2}{\text{Weight of brain tissue}\;\left(g\right)}$$

where GC1 and GC2 are glucose concentrations (mg/dL) before and after the incubation, respectively.

### 2.6 Preparation of Tissue Homogenate

The brain tissue was weighed and then chopped into 0.3 g pieces. The sliced tissues were homogenized using an electronic homogenizer in 3 mL of 50 mM ice‐cold homogenization buffer. After homogenization, the samples were centrifuged at 15000 rpm in a microcentrifuge programmed at 4°C for 15 min. For further ex vivo analysis, 2 mL of the supernatant was transferred into microtubes and subsequently stored at −80°C.

### 2.7 Ex Vivo Antioxidant Activity Assay

#### 2.7.1 Assessment of Reduced Glutathione (GSH) Level

The Ellman's approach (1959) was employed to measure the GSH concentration in brain tissues incubated with the different treatments. The homogenate mixtures were subjected to precipitation with 10% trichloroacetic acid (TCA), followed by centrifugation at 25°C for 10 min at a speed of 2000 rpm. Subsequently, 80, 40, and 200 μL of the supernatant, 0.5 mM DTNB, and 0.2 M Na_3_ PO_4_ buffer (pH 7.8) were added to 96 microwell plates. The absorbance was subsequently measured at a wavelength of 415 nm following a 15 min incubation period at 25°C.

#### 2.7.2 Assessment of Superoxide Dismutase (SOD) Level

According to Gee and Davison (1989), 0.1 mM DETAPAC solvent (170 μL) was transferred into a 96‐well plate, followed by the addition of 15 μL of either a sample or SOD test buffer. The samples were immediately mixed by applying gentle pressure to the plate on all sides after adding 15 μL of 1.6 mM 6‐hydroxyldopamine buffer. The absorbance was measured at a wavelength of 492 nm per min for 5 min.

#### 2.7.3 Assessment of CAT Activity Level

The tissue homogenate samples were analyzed using a previously established procedure for detecting lower absorbance of tissues in tests induced by H_2_O_2_ (Aebi 1984). By introducing 150 μL of 2 M H_2_O_2_ to a mixture containing 10 μL of homogenate and 340 μL of 50 mM Na_3_PO_4_ buffer at pH 7. The absorbance measurement was performed at a duration of 3 min, with readings collected at one‐minute intervals at a wavelength of 240 nm.

#### 2.7.4 Assessment of Malondialdehyde (MDA) Level

Malondialdehyde (MDA) concentration was determined as an index of lipid peroxidation using a modified thiobarbituric acid reactive substances (TBARS) assay (Chowdhury and Soulsby 2002). Briefly, 200 μL of the supernatant was mixed with 750 μL of acetic acid (20% v/v), 200 μL of sodium dodecyl sulfate (8.1%), 2 mL of thiobarbituric acid (0.25%), and 850 μL of distilled water. The reaction mixture was heated in a boiling water bath for 1 h, cooled to room temperature, and the absorbance was measured at 532 nm. The MDA concentration was used as an indicator of lipid peroxidation.

#### 2.7.5 Assessment of Nitric Oxide (NO) Level

The NO level in brain tissues was assessed using the Griess method, as previously reported (Erukainure et al. 2019). In brief, the supernatant (100 μL) or the blank (distilled water) was incubated for 30 min with an equal amount of Griess reagent in a dark environment at 25°C. Subsequently, the absorbance at a wavelength of 548 nm was measured for each sample.

### 2.8 Glycolysis Assays

#### 2.8.1 Assessment of Total Glycogen Content

With minor modifications, the glycogen content of the brain homogenate was estimated using a previously reported approach (Oyebode et al. 2022). A total of 0.3 g of tissue from the brain was liquefied in 300 μL of 30% KOH saturated in Na_3_PO_4_. This mixture was then heated for 30 min before being totally submerged in ice. To guarantee sufficient glycogen precipitation, the mixture was treated with 670 μL of 95% alcohol and centrifuged twice for 30 min. After draining the supernatant, the collected precipitate was dissolved in 1 mL of Milli‐Q H_2_O. Subsequently, an aliquot of 20 μL of the dissolved glycogen was mixed with 180 μL purified H_2_O, to which 200 μL of phenol (5%) and 1 mL of highly concentrated H_2_SO_4_ were added carefully. This mixture was vortexed gently before being heated for 10 min. After cooling, the absorbance was measured at 490 nm.

#### 2.8.2 Assessment of Glycogen Phosphorylase Level

Tissue samples (100 μL) were incubated for 10 min at 30°C with 4% glycogen and 64 mM glucose‐1‐phosphate. This reaction was stopped by adding 20% ammonium molybdate to a high‐concentration H_2_SO_4_. Subsequently, Elon reducer and distilled water were added to the reaction mixture, followed by incubation at 30°C for 45 min. Finally, the absorbance was measured at 340 nm.

#### 2.8.3 Assessment of Fructose 1,6 Bisphosphatase Level

A mixture of the supernatant (100 μL) and an equivalent amount of 0.1 M KCl and 0.05 M fructose, 1200 μL of 0.1 M Tris–HCl buffer, pH 7.0, 250 μL 1 mM EDTA, and 250 μL of 0.1 M MgCl_2_ was incubated for 15 min at 37°C. After stopping the enzyme action with 10% TCA, the mixture was centrifuged at 3000 rpm (4°C) for 10 min. Then, 100 μL of the supernatant was added to 50 μL of 1.25% ammonium molybdate and a freshly prepared 9% ascorbic acid solution. This was left to equilibrate for 20 min. Then, the absorbance was determined at 680 nm.

#### 2.8.4 Assessment of ATPase Level

At 37°C, a combination of equal quantities of tissue supernatant and solution of 5 mM KCl, 1.3 mL of 0.1 M Tris–HCl buffer, and 0.04 mL of 50 mM ATP were equilibrated for 30 min. Consequently, the reaction was stopped by adding 1 mL of Milli‐Q H_2_O and ammonium molybdate. The solution was allowed to cool down on ice for 10 min after mixing with freshly prepared 9% ascorbic acid. The sample's absorbance was obtained at 660 nm wavelength.

### 2.9 Assessment of AChE Activity

Using Ellman's protocol, the AChE activity of the supernatants was determined (Ellman 1959). In brief, 0.02 mL of the supernatant was combined with 0.01 mL of 3.3 mM Ellman's reagent, pH 7.0, and equilibrated for 20 min at 25°C with 0.05 mL of 100 mM phosphate buffer, pH 8. After adding 0.01 mL of 50 mM cholinergic iodide to the mixture, the absorbance at 412 nm was measured at 3‐min intervals.

### 2.10 ADME Properties of Phytochemicals Identified in Ethiopian Coffee Bean

The Simplified Molecular Input Line Entry System (SMILE) structures of compounds, including caffeic acid, caffeine, CGA, quinic acid, cafestol, kahweol, ferulic acid and catechol, identified previously from the Ethiopian coffee bean (Mohamed et al. 2024), were retrieved from the PubChem database. Then, the SMILE structures were uploaded to the SwissADME web tool at http://www.swissadme.ch/ to predict their physicochemical properties, pharmacokinetics, and drug‐likeness.

### 2.11 Molecular Docking

Computational docking studies were initiated to evaluate the binding interactions between compounds from Ethiopian coffee beans and the selected target enzymes, including CAT, SOD, and AChE. Ligand preparation involved structural optimization for correct hybridization and geometry using ChemAxon's MarvinSketch (V6.2.1) and Molegro Molecular Viewer. The enzyme structures for CAT (PDB: 1DGF; Wu and Dong 2012), SOD (PDB: 2C9V; Rikihisa 2017), and AChE (PDB: 4EY7; Dileep et al. 2022) were retrieved from the RCSB Protein Data Bank and preprocessed using Schrödinger Maestro (2023–2). The protein preparation protocol included multiple refinement steps: (1) assignment of proper bond orders and addition of hydrogen atoms, (2) removal of non‐essential water molecules (> 5 Å from binding site) and heteroatoms (except Ca^2+^ and Cl^−^ ions), (3) establishment of disulfide bridges and metal coordination bonds, and (4) generation of protonation states at physiological pH (7.0 ± 3). The most stable conformation was selected based on energy minimization using the OPLS_2005 force field, with convergence criteria set at RMSD < 0.30 Å relative to the crystal structure. Hydrogen bond networks were optimized using PROPKA at pH 7.0. For docking simulations, receptor grids were centered on the native ligand's binding site using Glide's grid generation module. High‐precision docking was performed in extra‐precision (XP) mode to accurately predict binding conformations and affinity scores (Nada et al. 2024). Resultant protein‐ligand complexes were visualized and analyzed in BIOVIA Discovery Studio to characterize key molecular interactions and binding energetics.

### 2.12 Statistics

All data were generated in triplicate and presented as mean ± SD. One‐way analysis of variance (ANOVA) was employed to detect significance differences between experimental groups at *p* < 0.05 using IBM's Statistical Package for the Social Sciences (SPSS) for Windows (Version 27.0). A Tukey multiple‐comparison test follows this analysis to determine differences between the various treatment groups and the control or untreated groups.

## 3 Results

### 3.1 Glucose Uptake in Rat Brain

**FIGURE 1:**
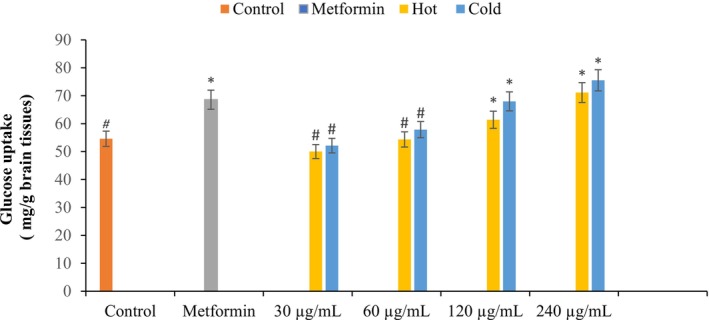
Effect of Ethiopian coffee bean extracts on the glucose uptake in rats' brain. Values = mean ± SD; *n* = 3. *Significantly different from untreated and #Significantly different from normal (*p* < 0.05, Tukey's HSD‐multiple range post hoc test, IBM SPSS for Windows).

Glucose uptake was significantly (*p* < 0.05) increased in the brain homogenate sample treated with higher concentrations of the Ethiopian coffee extracts (Figure 1) relative to the control treatment. While there was a 23% increase in brain glucose uptake for the hot coffee extract at 240 μg/mL compared to the control, a similar increase for the cold extract was approximately 28%. Overall, brain tissues treated with hot and cold plant extracts at 120–240 μg/mL had glucose uptake levels that were statistically comparable to those treated with metformin.

### 3.2 Antioxidative Activity

**FIGURE 2:**
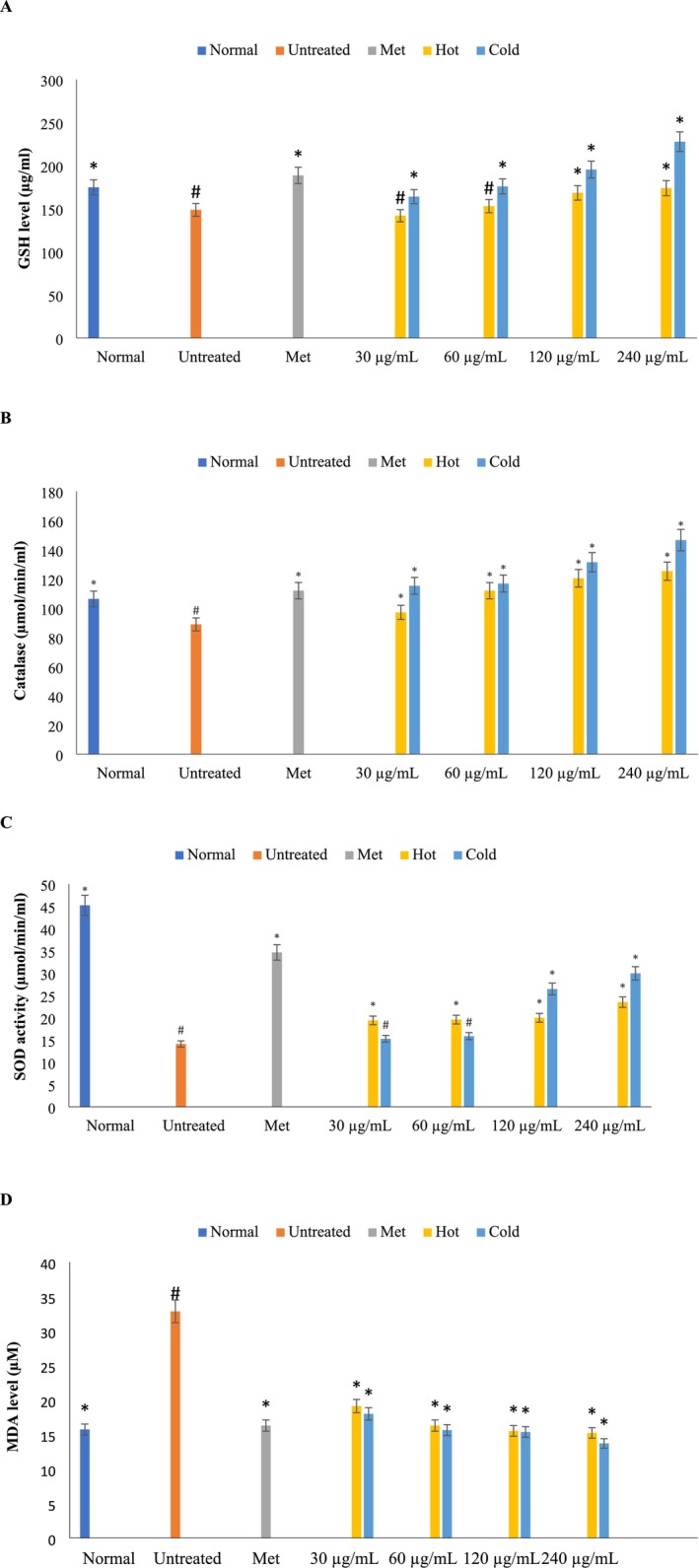
Effect of Ethiopian coffee bean extracts on (A) GSH concentration, (B) CAT activity, (C) SOD activity, and (D) MDA level in oxidative brain injury. Values = mean ± SD; *n* = 3. *Significantly different from untreated and #Significantly different from normal (*p* < 0.05, Tukey's HSD‐multiple range post hoc test, IBM SPSS for Windows).

As shown in Figure 2A–D, incubating brain tissue with glucose resulted in a significant (*p* < 0.05) decrease in GSH, CAT, and SOD levels while increasing MDA levels. The administration of both hot and cold coffee extracts resulted in a significant (*p* < 0.05) improvement in GSH, SOD, and CAT levels, as well as a reduction in MDA levels. Higher concentrations of the cold coffee extract, at 120–240 μg/mL, increased SOD activity more effectively compared to metformin. Overall, the cold coffee extract exhibited better antioxidant activities than the hot extract.

**FIGURE 3:**
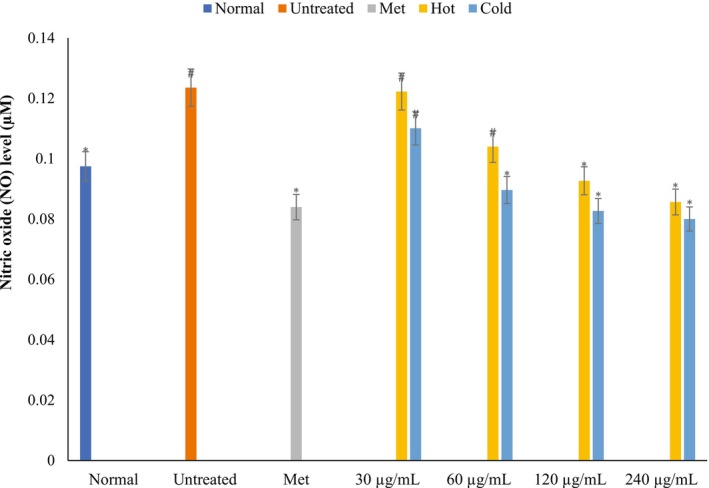
Effect of Ethiopian coffee bean extracts on NO activity level in oxidative brain injury. Values = mean ± SD; *n* = 3. *Significantly different from untreated and #Significantly different from normal (*p* < 0.05, Tukey's HSD‐multiple range post hoc test, IBM SPSS for Windows).

In Figure 3, brain NO levels in the untreated brain tissue were significantly (*p* < 0.05) elevated after hyperglycemia induction. However, after treatment with the Ethiopian coffee extracts, NO levels were dramatically reduced, especially at higher concentrations (120–240 μg/mL). Furthermore, the cold extract reduced NO in the plant treatment groups to normal tissue levels better than the hot extract sample.

### 3.3 Glycolytic Enzymes' Activity

**FIGURE 4:**
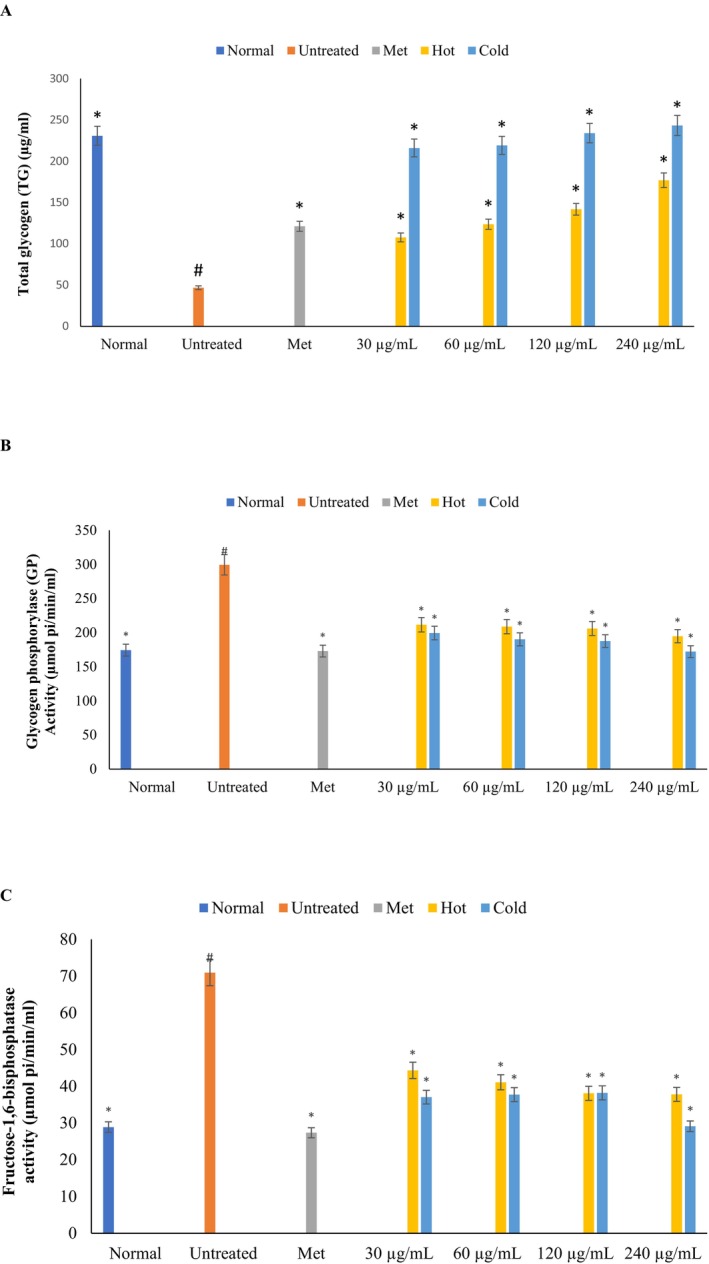
Effect of Ethiopian coffee bean extracts on (A) TG, (B) GP, and (C) Fructose‐1,6‐bisphosphatase activity level in oxidative brain injury. Values = mean ± SD; *n* = 3. *Significantly different from untreated and #significantly different from normal (*p* < 0.05, Tukey's HSD‐multiple range post hoc test, IBM SPSS for Windows).

After co‐incubation with glucose, the untreated animals' brain fructose‐1,6‐bisphosphatase and glycogen phosphorylase activities increased considerably while glycogen content decreased in the same group (Figure 4A–C). Treatment with various coffee extracts increased the activities of the former enzymes. Although the capacities of these extracts to lower these enzyme activities are not statistically different at the test concentrations, the cold extract improved glycogen content in brain tissue more than the hot extract.

### 3.4 Assessment of ATPase Activity

**FIGURE 5:**
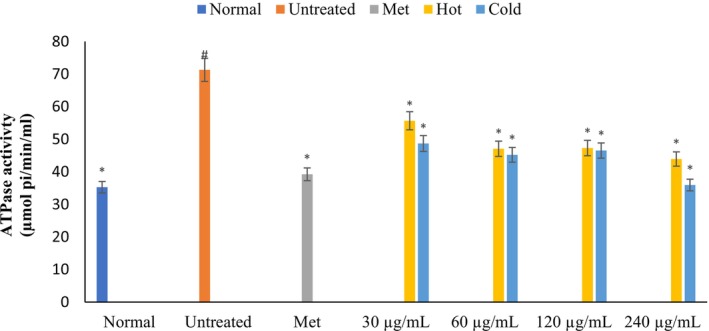
Effect of Ethiopian coffee bean extracts on ATPase activity level in oxidative brain injury. Values = mean ± SD; *n* = 3. *Significantly different from untreated and #Significantly different from normal (*p* < 0.05, Tukey's HSD‐multiple range post hoc test, IBM SPSS for Windows).

Incubation with glucose only significantly (*p* < 0.05) increased the purinergic activity of the brain injury, as shown by elevated ATPase activity, as displayed in Figure 5. However, following treatment with Ethiopian coffee, these levels were significantly (*p* < 0.05) reduced in a dose‐dependent manner. The effect of cold coffee extract on ATPase levels is similar to that of high concentrations of coffee, as observed in normal tissue and in the presence of metformin.

### 3.5 Assessment of AChE Activity

**FIGURE 6:**
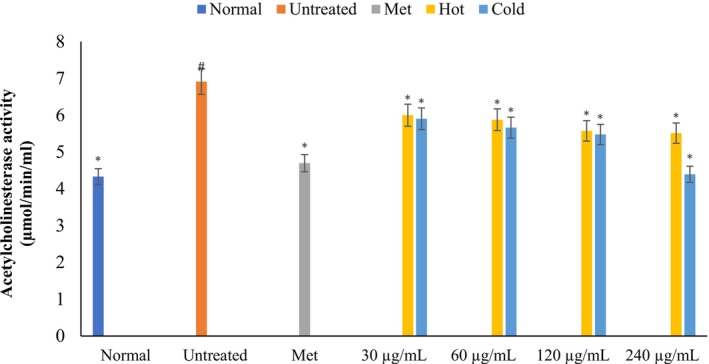
Effect of Ethiopian coffee bean extracts on AChE activity level in oxidative brain injury. Values = mean ± SD; *n* = 3. *Significantly different from untreated and #Significantly different from normal (*p* < 0.05, Tukey's HSD multiple range post hoc test, IBM SPSS for Windows).

Data in Figure 6 showed that AChE activity increased significantly (*p* < 0.05) in brain tissue incubated with only glucose. However, the activity of this enzyme decreased significantly (*p* < 0.05) after treatment with the Ethiopian coffee extracts. The test concentrations of the coffee bean extract significantly (*p* < 0.05) lowered brain AChE activity to levels comparable to those of the normal and metformin experimental groups.

### 3.6 ADME Profile

The ADME‐predicted pharmacokinetic parameters suggest that caffeic acid, caffeine, dihydroferulic acid, gallic acid, patuletin, and theacrine exhibit high gastrointestinal absorption, as shown in Table 1. All the identified compounds were predicted to have no BBB permeability. Moreover, these compounds exhibit no inhibitory activity towards the CYP1A2, CYP2C19, CYP2C9, and CYP2D6 enzymes, and are free of any PAIN alerts.

**TABLE 1:** The ADME properties of the identified compounds in Ethiopian coffee beans ( *Coffea arabica* ).

Compounds	TPSA	Water solubility	Log O/W	Gl absorption	BBB permeant	CYP1A2 inhibitor	CYP2C19 inhibitor	CYP2C9 inhibitor	CYP2D6 inhibitor
Caffeic acid	77.76 Å^2^	Very soluble	0.93	High	No	No	No	No	No
Caffeine	61.82 Å^2^	Very soluble	−0.08	High	No	No	No	No	No
CGA	164.75 Å^2^	Very soluble	−0.39	Low	No	No	No	No	No
Quinic acid	66.76 Å^2^	Very soluble	1.31	High	No	No	No	No	No
Cafestol	97.99 Å^2^	Very soluble	0.21	High	No	No	No	No	No
Kahweol	140.59 Å^2^	Soluble	1.38	Low	No	No	Yes	No	No
Ferulic acid	70.93 Å^2^	Very soluble	−0.16	High	No	No	No	No	No

### 3.7 Molecular Docking Analysis

**FIGURE 7:**
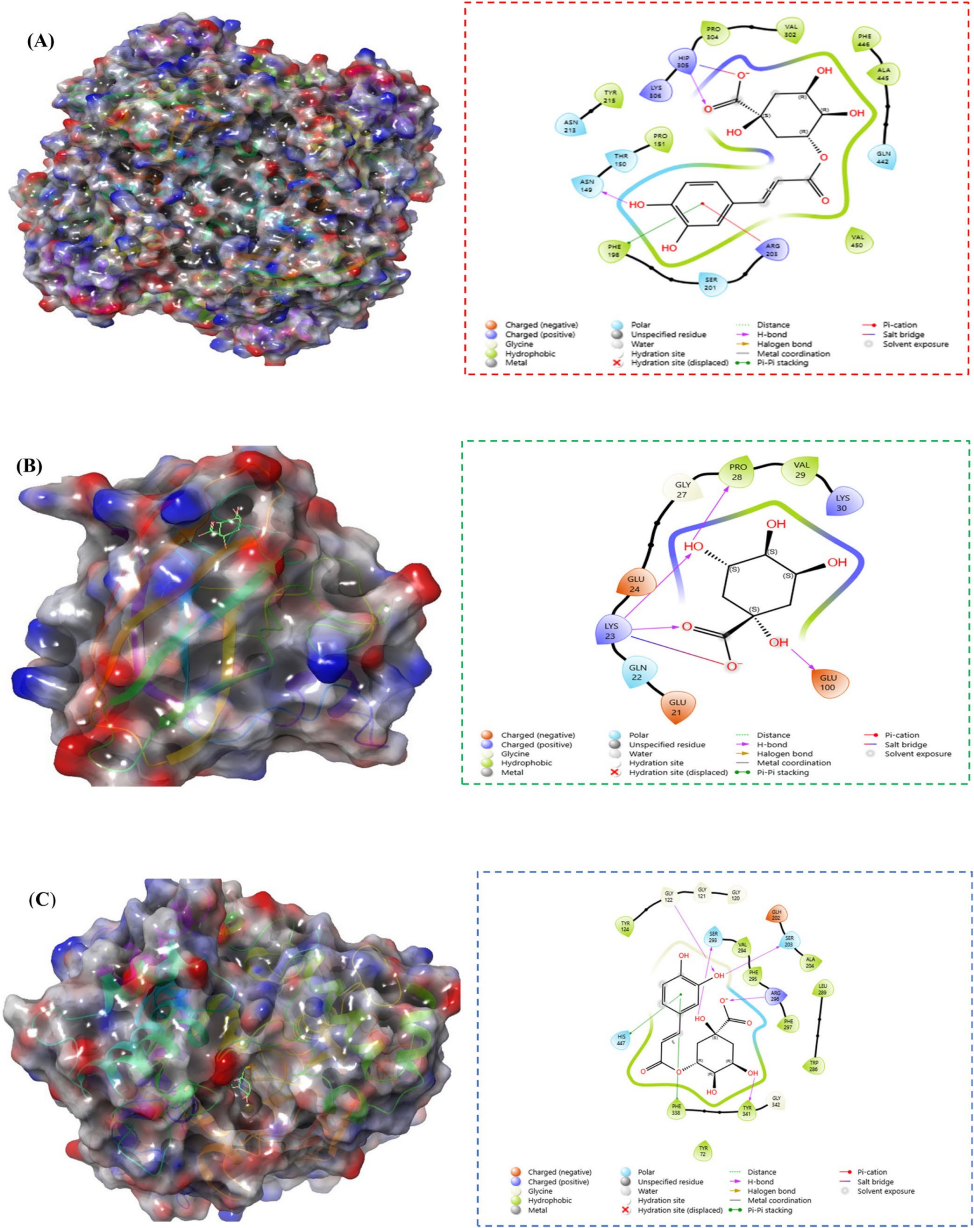
3D and 2D interactions of (A) CGA_CAT, (B) quinic acid_SOD, and (C) CGA_AChE complexes.

Molecular docking was used to evaluate the interactions of the compounds identified in Ethiopian coffee beans with the enzymes CAT, SOD, and AChE. The binding free energies for these interactions are presented in Figure 7A–C and Table 2. Among the compounds, CGA exhibited the strongest binding affinity for CAT (−6.08 kcal/mol) and AChE (−7.06 kcal/mol), whereas quinic acid showed the strongest affinity for SOD (−5.29 kcal/mol). Overall, the binding energies for all compounds ranged from −7.06 to −1.89 kcal/mol (Table 2).

**TABLE 2:** The binding energy of Ethiopian coffee beans identified compounds with selected enzyme targets.

Compounds	Free binding energy (kcal/mol)		
	CAT	SOD	AChE
Caffeic acid	−4.56	−4.66	−5.07
Caffeine	−5.25	−4.74	−6.75
CGA	−6.08	−3.08	−7.06
Quinic acid	−5.48	−5.29	−6.20
Cafestol	−4.29	−2.10	−6.18
Kahweol	−4.22	−1.89	−7.04
Ferulic acid	−4.53	−3.88	−4.86
Catechol	−5.65	−4.50	−5.43

## 4 Discussion

Poor facilitative glucose transport across the BBB due to impaired glucose transporter function in T2D has been linked to neurodegeneration (Verdile et al. 2015). Moreover, other studies suggest that impaired glucose uptake in the brain is associated with progressive neuronal dysfunction (Daulatzai 2017; Duarte 2015). This condition is linked to oxidative stress arising from dysregulated glucose metabolism. Although there is existing evidence that suggests coffee beans have anti‐inflammatory and antioxidant properties (Hu et al. 2019), this study, for the first time, investigated the impact of Ethiopian coffee bean extract (hot and cold) on glucose uptake and its modulatory influence on glucose‐induced oxidative imbalance in isolated rat brain tissue using ex vivo experimental analysis. Although this investigative model lacks the physiological complexity that exists in normal tissue conditions, its outcome provides better reliability than in vitro cell models.

Several studies have shown that inadequate energy metabolism in neuropathy patients is mediated by impaired brain glucose uptake, particularly by neurons (Lalić et al. 2008; Powers et al. 2007).

Researchers have also found that increased glucose flow through the brain enhances cognitive abilities (Peters et al. 2020). Based on our results (Figure 1), increased glucose uptake in brain tissue after incubation with Ethiopian coffee indicates that coffee metabolites stimulate glucose uptake, thereby demonstrating improved uptake potential and, consequently, enhanced neuronal activation. The chemical profile of Ethiopian coffee beans ( *Coffea arabica* ) has previously been identified by Mohamed et al. (2024) to include various compounds, including CGA, caffeic acid, quinic acid, caffeine, cafestol, kahweol, ferulic acid, and catechol, none of which were detected using the cold method. However, several compounds, including caffeine and quinic acid, were found in both extracts (Mohamed et al. 2024). Interestingly, CGA, one of the metabolites identified in this plant, has been reported to cross the BBB and elicit physiological responses (Erickson and Banks 2018). Consequently, the high glucose uptake by the cold coffee extract at 120 and 240 μg/mL could be attributed to the CGA. CGA may provide neuroprotection by increasing BBB function and reducing brain damage (Heitman and Ingram 2017; Liu et al. 2022). The notable BBB permeability of CGA enhances its distribution in the brain, thus conferring a substantial advantage in neuroprotective mechanisms (Liu et al. 2022; Zeng et al. 2022). In contrast, based on the ADME analysis, the identified compounds were predicted to have limited BBB permeability. However, these predictions are derived from computational models and may not fully reflect in vivo transport and metabolic processes. Furthermore, the present study employed an ex vivo brain tissue model, in which neuroprotective effects were assessed directly on brain tissue and therefore did not depend on BBB penetration. Consequently, further pharmacokinetic studies are needed to confirm the brain bioavailability of coffee‐derived phytochemicals and their metabolites.

Prolonged hyperglycemia‐induced oxidative stress has been identified as one of the contributory factors of neurodegeneration in T2D (Lima et al. 2022). Excess ROS generation, which may injure brain macromolecules such as lipids, proteins, and DNA, is called oxidative stress (Bhat et al. 2015). The brain is particularly susceptible to oxidative stress, as it is one of the most metabolically active organs in the body (Rodriguez‐Rodriguez et al. 2014). Indeed, it has been noted that decreased levels of antioxidant enzymes have been identified as a significant factor contributing to the development of brain degeneration (Salau et al. 2021). According to our findings, the decrease of GSH, CAT, and SOD activities after glucose incubation in Figure 2a–c indicates the existence of oxidative stress in brain tissues, which could be related to hyperglycemia‐induced ROS production. This metabolic condition may lead to an increase in brain lipid peroxidation. However, the elevation of GSH, CAT, SOD, and the reduction of MDA following the treatment of Ethiopian coffee suggests that it may have a potential neuroprotection property. These antioxidant effects could be supported by strong affinities of phenolic compounds such as CGA and quinic acid in the coffee with the active site amino acid of CAT and SOD during molecular docking analysis (Figure 7A,B).

Previous research has demonstrated the role of nitric oxide in regulating blood flow and signaling in the brain (Picón‐Pagès et al. 2019). NO is produced by the enzyme NO synthase (NOS), which has different isoforms, including the cytokine‐inducible (iNOS), neuronal (nNOS), and endothelial (eNOS) types. These isoforms function in generating NO from L‐arginine (Schulz et al. 2005). The increased NO level in brain tissues after glucose incubation (Figure 3) may suggest a proinflammatory effect. Oxidative tissue damage is capable of recruiting immune cells, which ultimately activate pathways that lead to elevated iNOS activity. As this enzyme produces sustained levels of NO, more of the signaling molecule interacts with superoxide radicals to form peroxynitrite anion (Radi et al. 1991). This generated radical thus exacerbates the oxidative imbalance that worsens altered biochemical homeostasis in the tissue, thus contributing to neurotoxicity. However, the decrease in NO level seen in groups exposed to Ethiopian coffee bean treatment may suggest possible therapeutic advantages in reducing nitrosative stress in the brain.

Glycolysis is a metabolic pathway that regulates glucose metabolism and stimulates insulin secretion, thereby controlling the physiological activities of various cells (Guo et al. 2012). It occurs in the normal human brain and elevates normally with increasing neurological activity (Adeva‐Andany et al. 2014). Every cell undergoes glycolytic processes catalyzed by enzymes (Rizzieri et al. 2019). Glycogen and glycogen phosphorylase are essential for glycogenolysis, whereas fructose‐1,6‐bisphosphate is essential for gluconeogenesis (Salau et al. 2020). However, inhibiting these enzymes is effective in treating and controlling type 2 diabetes (T2D) (Hayes et al. 2014). The enhanced glycogen phosphorylase activity in brain tissues incubated with glucose in Figure 4b could imply an increased breakdown of brain glycogen to glucose.

Similarly, increased fructose‐1,6‐bisphosphatase activity inhibits glycolytic flux in glucose‐incubated brain regions (Figure 4c), indicating greater glucose production. The presence of glycogenolysis and gluconeogenesis in the brain can increase glucose production at a considerable rate. However, the decreased activity of these enzymes after being subjected to Ethiopian coffee beans may indicate the activation of glycogenesis and glycolysis, which is corroborated by increased brain glycogen content (Figure 4a). Consequently, glucose concentrations are lowered, and ROS is depleted, suggesting that Ethiopian coffee beans can modify impaired glucose metabolism in neurodegenerative disorders.

Adenosine is an essential neuroactive nucleoside that regulates cellular homeostasis. It is primarily generated by ATP breakdown, which occurs under anoxic conditions (Barsotti and Ipata 2004). Adenosine regulates synaptic plasticity, immature neural cell differentiation, and proliferation, whereas ATP is associated with presynaptic neuromodulation and other critical brain functions (Rimbert et al. 2023). However, Van De Wal et al. (2022) documented that the depleted ATP levels were associated with neurodegenerative disorders in *Ndufs4* knockout mouse models. In our results, ATPase activity in the brain (Figure 5) increased after incubation with glucose, suggesting a decrease in brain ATP adenosine levels. This finding is consistent with prior research by Olofinsan et al. (2022), which showed that a disruption of glucose transport in isolated rat brains leads to abnormal activation of purinergic enzymes. However, the restored ATPase activity in the brain after the coffee treatment suggests that this plant product plays a valuable role in improving brain energy homeostasis.

Acetylcholinesterase (AChE), the brain's principal cholinesterase, hydrolyzes ACh to choline and acetate (Colovic et al. 2013). AChE is produced exclusively at neuromuscular junctions and became one of the initially excitatory neurotransmitters recognized (Colombo and Francolini 2019). Despite efforts to establish effective ways to manage brain illnesses such as Alzheimer's disease, clinical studies have shown that AChE inhibitors propose therapeutic advantages by exerting anti‐neurodegenerative properties (Moss and Perez 2021). Studies by Salau et al. (2021) showed that an elevation in AChE levels in brain tissue was linked with oxidative stress. The observed reduction in AChE activity at high concentrations of coffee bean extract, as shown in Figure 6, suggests a potent neuroprotective effect. Notably, the degree of AChE inhibition achieved by the coffee extract was greater than that of the anti‐diabetic drug metformin, which was used as a reference control in this assay. Although a study by Falade et al. (2025) revealed that donepezil inhibits AChE with an IC_50_ of 0.008 μg/mL in a similar ex vivo brain model experiment, another study reported that metformin may be a potential therapeutic option for dementia, particularly in patients with T2D (Cui et al. 2024). The inhibitory effect of the coffee bean, as observed in Figure 6, was supported by the in silico results in Figure 7C and Table 2, which show that the CGA, one of the constituents in the coffee extract, interacts strongly with the AChE protein. While donepezil in the work by Falade et al. (2025) had a binding affinity of −9.4 with AChE, CGA in the present investigation had lower binding energy (−7.06 kcal/mol) which still suggests thermodynamically favorable interaction. The presence and location of benzoic acid functional groups on aromatic ring structures and their derivatives have been documented to influence the bioactivity of various compounds (Işık and Beydemir 2021). Thus, the effectiveness of CGA on AChE may be associated with its chemical structure, which could be linked to its pharmacological activity.

### 4.1 Study Limitations

The ex vivo glucose‐exposed brain model primarily reproduces acute metabolic and oxidative stress rather than the full complexity of chronic diabetic neuropathy. The model was selected as a controlled system for evaluating direct neuroprotective and antioxidant effects of coffee extracts on brain tissue. Therefore, our findings should be considered preliminary and require confirmation in appropriate in vivo diabetic models.

## 5 Conclusion

Findings from this study suggest that Ethiopian coffee beans ( *Coffea arabica* ) may possess the potential biological activity to prevent hyperglycemia‐induced brain oxidative damage. This is due to its extracts' ability to mitigate brain damage and modulate purinergic and cholinergic dysfunctions while improving altered glucose metabolism in brain tissues. The characterization of the cold and hot coffee extracts revealed the presence of various chemical components that could be linked to these observations. Interestingly, the cold coffee extract had a greater concentration of bioactive components and demonstrated higher activity compared to the hot coffee extract. The in silico analysis also highlighted that phenolic acids, such as CGA, among the compounds found in the coffee extracts, demonstrated a stronger interaction with all enzymes under study, with a good pharmacological profile and bioavailability. Nevertheless, in vivo and molecular investigations are required to elucidate how coffee compounds ameliorate hyperglycemia‐induced neurodegeneration. The outcomes of these results will contribute to the development of innovative therapies for diabetes‐related brain disorders.

## Author Contributions

**Huda Ismail:** investigation, writing – review and editing, visualization, methodology. **Almahi I. Mohamed:** conceptualization, investigation, methodology, validation, writing – review and editing, writing – original draft, software, data curation, formal analysis, visualization. **Md. Shahidul Islam:** writing – review and editing, project administration, supervision, funding acquisition. **Kolawole A. Olofinsan:** methodology, visualization, data curation, writing – review and editing. **Ochuko L. Erukainure:** investigation, writing – review and editing, data curation.

## Funding

This study has been supported by Incentive Funding for Rated Researchers from the National Research Foundation (NRF), Pretoria; and Research Reward from the Research Office of the University of KwaZulu‐Natal, Durban, South Africa.

## Conflicts of Interest

The authors declare no conflicts of interest.

## Data Availability

The data that support the findings of this study are available from the corresponding author upon reasonable request.
